# Natural Aging of Reprocessed Polypropylene Composites Filled with Sustainable Corn Fibers

**DOI:** 10.3390/polym16131788

**Published:** 2024-06-25

**Authors:** Antonio Zilverlan Germano Matos, Alisson Rodrigues de Oliveira Dias, Ana Carolina Ferreira dos Santos Rosa, Renato de Sousa Nascimento Junior, Cristiano José de Farias Braz, Lucas Rafael Carneiro da Silva, Amanda Dantas de Oliveira, Renata Barbosa, Tatianny Soares Alves

**Affiliations:** 1 Postgraduate Program in Materials Science and Engineering, Technology Center, Federal University of Piauí, Teresina 64049-550, PI, Brazil; zilverlan@gmail.com (A.Z.G.M.); rodrigues_alisson@live.com (A.R.d.O.D.); renatosousa705@hotmail.com (R.d.S.N.J.); cristiano.farias@gmail.com (C.J.d.F.B.); rrenatabarbosa@yahoo.com (R.B.); 2 Materials Engineering Course, Technology Center, Federal University of Piauí, Teresina 64049-550, PI, Brazil; carol_fsr@outlook.com; 3 Postgraduate Program in Mining, Metallurgical and Materials Engineering, Federal University of Rio Grande do Sul, Porto Alegre 91501-970, RS, Brazil; lr.rafaelcarneiro@gmail.com; 4 Postgraduate Program in Materials Science and Engineering, Federal University of Pelotas, Pelotas 96010-610, RS, Brazil; amandaoliveira82@gmail.com

**Keywords:** corn husk fiber, mechanical properties, morphology analysis, reprocessed polypropylene composites

## Abstract

Natural fiber reinforcements have the potential to enhance mechanical properties, thereby improving performance and durability in various applications. In this study, we comprehensively evaluated the impact of environmental degradation over 120 days on reprocessed polypropylene (PP) reinforced with corn husk fiber (CHF) composites. The manufactured systems underwent rigorous analysis using various techniques, including Fourier transform infrared spectroscopy, thermogravimetric analysis, optical microscopy, scanning electron microscopy, and tensile testing. These analyses revealed that climatic conditions significantly influenced (*p* < 0.05) the mechanical properties of all systems. Photodegradation led to surface morphological changes and chemical structures. Regardless, adding CHF filler proved a key factor, as it allowed for less susceptibility to environmental degradation than the reprocessed matrix. These findings, therefore, provide robust evidence supporting the feasibility of using CHF composites for manufacturing agricultural containers.

## 1. Introduction

Polypropylene (PP) is a highly adaptable thermoplastic polymer widely recognized for its excellent heat, moisture, and chemical resistance as well as its favorable processing characteristics and recyclability [1,2]. Despite being subject to degradation phenomena like photooxidation, which can impact its elongation at break and impact strength [3,4], polypropylene’s mechanical behavior, including its stress–strain curves, can be effectively controlled by strain rate and temperature [5]. This adaptability allows PP to be utilized in various electronics, electrical, automotive, textile, and pipeline applications [6,7].

Polymer composites must combine natural fiber reinforcements and recyclable polymer matrixes [8,9,10,11]. Natural fiber reinforcements improve strength and stiffness, enhancing performance and durability in various applications [12,13]. However, their high hygroscopicity limits their usefulness in high-performance structural applications due to compromised durability [14,15]. Understanding the long-term behavior of these composites requires attention, as both natural fiber reinforcements and reprocessed thermoplastic systems are more susceptible to environmental aging than virgin synthetic composites [16,17]. Long-term performance assessment is crucial for adopting these materials in engineering applications.

Kuram [18] reported recent advances in the field of natural fibers, with a focus on green composites derived from various agricultural waste fibers such as almond husk, areca nut husk, argan nut husk, bambara nut husk, wheat husk, corn, cotton shell, hazelnut shell, macadamia shell, olive kernel, orange peel, pineapple leaf, pistachio shell, potato shell, pumpkin seed shell, rice shell, sunflower shell, walnut, wheat bran, and yerba mate. The results indicated that composites made from these agricultural waste fibers could gain wider acceptance and that developing new value-added applications for these composites would offer more cost-effective materials for the international market.

Fajardo Cabrera de Lima et al. [19] evaluated the effect of natural aging on PP and bamboo fiber composites’ mechanical, physical, and thermal properties. They reported that all exposed composites suffered a decrease in their properties. Reprocessed polymer composites reinforced with corn husk fiber (CHF) could improve their performance during natural aging. CHF has been used as reinforcement in bio-polyethylene composites and has shown that interface strength and fiber content can influence the stiffness of these materials [20]. Additionally, Tarrés and Ardanuy [21] used cellulose fibers and corn husk in bio-polyethylene-based composites, which showed significant results in their thermomechanical properties. These results highlight the following research hypothesis: using CHF as reinforcement in reinforced reprocessed polymers could improve their performance during natural aging.

Using agricultural residues such as CHF to produce sustainable polymer composites is promising [22,23]. Due to its low cost and abundance, incorporating CHF into PP composites could enhance thermal stability, mechanical properties, morphological characteristics, and economic benefits [22,24]. This would offer a sustainable solution by utilizing agro-waste, thus aligning with principles of green chemistry and contributing to the development of biodegradable materials [25]. The combination of these fibers with sustainable polymer matrices has the potential to ensure circularity and deal with end-of-life composite waste, promoting sustainability and contributing to the circular economy [25,26,27].

In the agricultural sector, polymer-based components are exposed to harsh environmental conditions such as sunlight and heat [28]. These materials will inevitably undergo significant changes in their properties over time [19,29]. This study evaluated the effect of natural aging over 120 days on the properties of reprocessed PP composites reinforced with CHF, enabling their potential use as agricultural containers.

## 2. Materials and Methods

### 2.1. Materials

PP H 301 (Braskem, Camaçari, Brazil), a polymer recommended for injection molding, was used as a polymer matrix. The polymer has a density of 0.905 g/cm^3^ (ASTM D792) [30] and a melt flow index (MFI) of 10 g/10 min (230 °C/2.16 kg) (ASTM D1238) [31]. The Camaloura community (Castelo do Piauí, Brazil) donated the CHF, which was incorporated into PP as an ecological filler.

### 2.2. Preparation of Composites and Specimens

**Figure 1:**
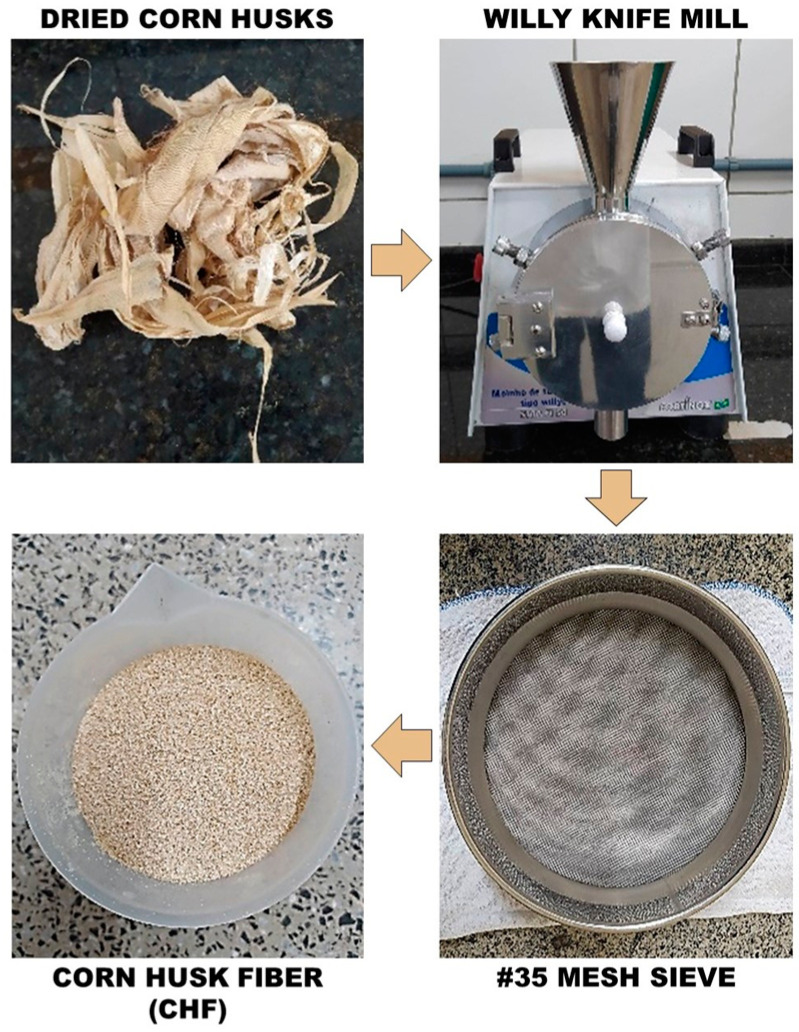
Treatment and processing of CHF.

Initially, the CHF was manually cleaned to remove impurities and dried in the sun for seven days. Then, the raw material was manually cleaned again and ground in a Willy knife mill (Model STAR FT-50, Fortinox, Brazil). The ground material was sieved through a 35 mesh (opening of 0.5 mm), and a powder of CHF was obtained. Figure 1 shows the treatment and processing of CHF after drying.

The PP was reprocessed in a single-screw extruder (L/D = 26) (Model AX-16, AX Plásticos, São Paulo, Brazil) once and thrice without incorporating CHF (control). The temperature profile in the three heating zones was 180, 190, and 200 °C, respectively, and the screw rotation speed was 50 rpm. Subsequently, the PP, the ground CHF, and the additives were dried in an oven at 80 °C for 24 h, and the composites were prepared as described below. All components of the formulation were mixed manually before processing. The compositions were processed in an extruder under the same conditions presented for neat PP. The CHF was introduced into the extruder only during the final reprocessing cycle.

After reprocessing the compositions (dry and pelletized material) in the extruder, specimens were produced using an injection mold (Model BL52, Eurostec, Caxias do Sul, Brazil) for tensile testing, TYPE I, according to ASTM D638 [32]. The temperature profile used in the injection cylinder was 205, 200, and 190 °C, in the injection nozzle 185 °C, and in the mold 25 °C. The cooling time used was 25 s. As a result, different compositions were developed and evaluated, including reprocessed PP (R-PP) formulations subjected to one reprocessing cycle (R-PP1x) and three reprocessing cycles (R-PP3x), both with and without 3 wt% of CHF (R-PP3x/3CHF) and 5 wt% of CHF (R-PP3x/5CHF).

### 2.3. Natural Exposure Aging

The current study assessed the degradation inherent to different composite materials (R-PP1x, R-PP3x, R-PP3x/3CHF, R-PP3x/5CHF) under conditions conforming to ASTM D1435 [33]. According to previously reported adaptations [34], the samples were exposed for up to 120 days (between August and December 2022) using a device to maintain an inclination of 5° about the ground and always directed toward the Equator. The exposure site was in an open area within the UFPI (5°03′19.8″ S 42°48′00.3″ W).

### 2.4. Fourier Transform Infrared Spectroscopy (FTIR) Analysis

The evaluation of alterations in the chemical structure of polymers consequent to ultraviolet (UV) radiation exposure was performed utilizing an FTIR spectrophotometer (Model Spectrum 400, PerkinElmer, Shelton, CT, USA) coupled with the attenuated total reflectance (ATR). The analysis operated at wavenumbers between 4000 and 500 cm^−1^, with a 4 cm^−1^ resolution and transmittance mode. For this analysis, films of the composites were produced by thermo-compression.

### 2.5. Thermogravimetric Analysis (TGA)

The thermal stability of the composites was assessed using a thermogravimetric analyzer (Model TGA-51M, Shimadzu, Kyoto, Japan). Approximately 10 mg of each sample was heated from room temperature to 800 °C, with a heating rate of 10 °C/min under a nitrogen atmosphere and a 50 mL/min flow rate.

### 2.6. Tensile Test

The mechanical behavior in the tensile test was performed on a Universal Testing Machine (Model DL 30000, EMIC, São Paulo, Brazil) according to ASTM D638 [32], using a 5 kN load cell and a crosshead speed of 50 mm/min at room temperature. At least seven specimens of each competition were evaluated.

All measurements were statistically investigated by a three-factor (composition, temperature, and time) analysis of variance (ANOVA) using OriginPro^®^ 2022 software (Northampton, MA, USA). The significant difference between the means was evaluated using Tukey’s test with a 95% confidence level.

### 2.7. Morphology Analysis

The surface morphology of the samples aging before and after the tensile test was analyzed using a binocular optical microscope (Model DM500, Leica Microsystems, Wetzlar, Germany) in reflection mode with an ICC50 camera at 40× magnification. Scanning electron microscopy (SEM) was also used (Model Quanta 250 FEG, FEI, Hilsboro, OR, USA), with an accelerating voltage of 10 kV at 1.5 k× magnification. Previously, all surfaces were coated with a thin layer of gold using a metallizer (Model Q150R, Quorum, East Sussex, UK).

## 3. Results and Discussion

### 3.1. FTIR Analysis

**Figure 2:**
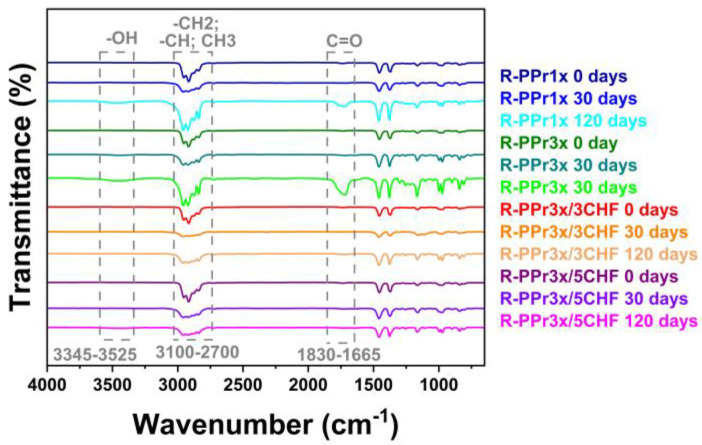
FTIR spectra of the systems (R-PP1x, R-PP3x, R-PP3x/3CHF, and R-PP3x/5CHF) before and after natural aging (0, 30, and 120 days).

FTIR spectroscopy is recognized and effective for observing changes in the chemical structure of polymers induced by exposure to UV light [34]. Figure 2 shows the infrared spectra of the materials under study before and after natural aging.

In all samples, the absorption bands in the 2955, 2920, and 2870 cm^−1^ regions are related to the stretching of the C–H groups. The symmetric and asymmetric deformation of the CH_2_ and CH_3_ groups at 1460 cm^−1^. The stretching of the C–C bonds of the isopropyl group at 1170 cm^−1^ and the angular deformation of the –CH groups manifests at 815 cm^−1^. This pattern is compatible with the structure of PP, which has been reported previously [35,36]. At 1735 cm^−1^, it is attributed to the reprocessing of PP, which produced carbonyl during matrix thermo-oxidation [37,38]. Ester absorption is observed at 1735 cm^−1^ and in α, β-unsaturated carbonyl species between 1700 and 1600 cm^−1^. In this case, only one peak was evident at 1660 cm^−1^. A band also appeared at 1620 cm^−1^, which could be attributed to the vinyl or conjugated vinyl groups [39].

Exposure to light induces the formation of free radicals in the polymer, breaking carbon–carbon or carbon–hydrogen bonds. Under oxidative conditions, this breakdown generates highly reactive peroxide radicals, which remove hydrogen atoms from neighboring molecules, forming hydroperoxide groups. In the presence of light, these groups become unstable, causing chain fragmentation and water production, carbonyl groups, and vinyl groups [40]. The reprocessed matrices showed the appearance of a band in the regions between 3345 and 3525 cm^−1^ and 1720 cm^−1^ that did not exist in the polymer and increased with increasing exposure time [41,42]. Badji et al. [43] examined the impacts of natural weathering on PP biocomposites reinforced with hemp fibers. They noted a strong correlation between sample whitening and the formation of C=C double bonds resulting from lignin degradation.

**Figure 3:**
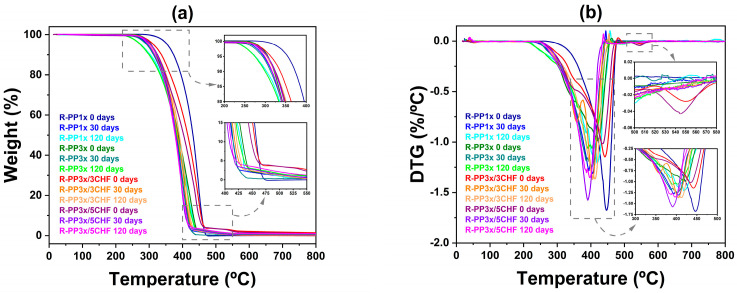
Thermograms of (**a**) TGA and (**b**) DTG of the systems (R-PP1x, R-PP3x, R-PP3x/3CHF, and R-PP3x/5CHF) before and after natural aging (0, 30, and 120 days).

**Figure 4:**
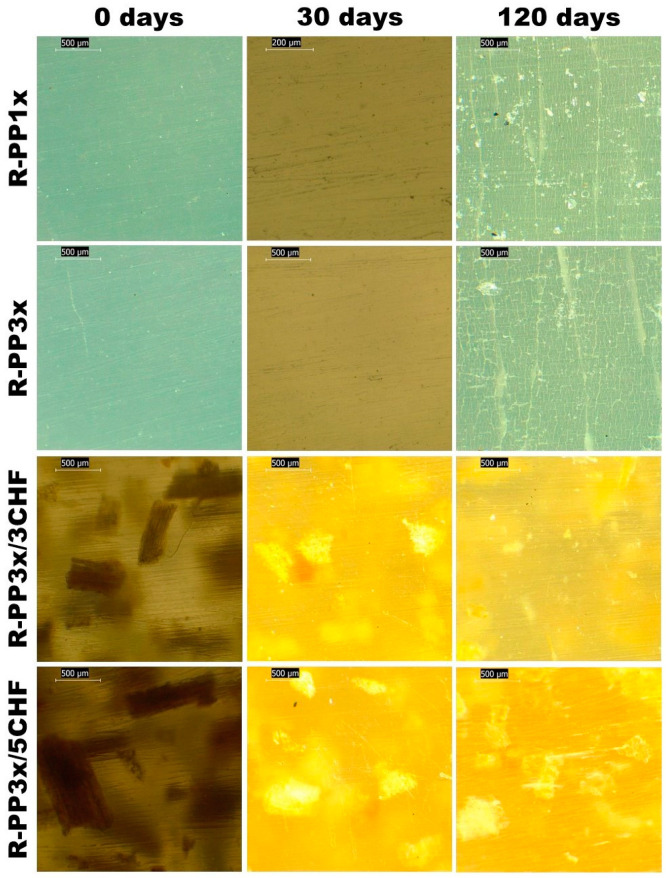
Optical microscopy of the sample surface of the systems (R-PP1x, R-PP3x, R-PP3x/3CHF, and R-PP3x/5CHF) before and after natural aging (0, 30, and 120 days) (40× magnification).

**Figure 5:**
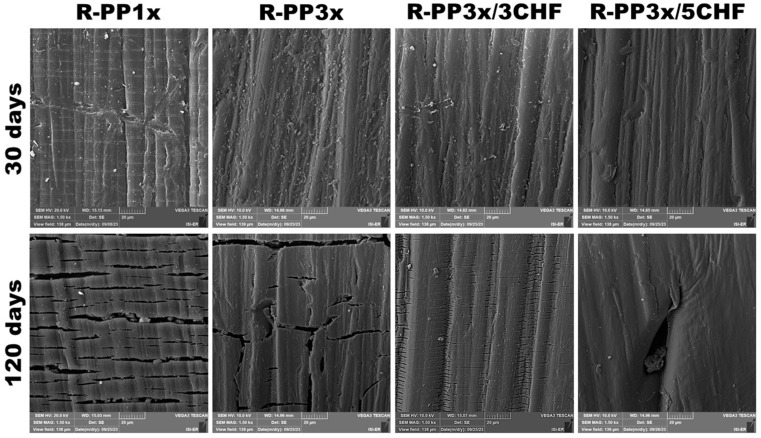
SEM of the sample surfaces of the systems (R-PP1x, R-PP3x, R-PP3x/3CHF, and R-PP3x/5CHF) after 30 and 120 days of natural aging (1.5 k× magnification).

The presence of these bands indicates the formation of hydroxyls (free –OH or carboxylic acid groups) and carbonyls [44], confirming the occurrence of degradation in polymer samples reprocessed after 120 days of exposure. However, in composites, the increase in the intensity of this band was subtle, suggesting that degradation in these materials was attenuated, which is in accordance with observations from TGA (Figure 3) and images obtained by optical microscopy (OM) and SEM (Figure 4 and Figure 5). Photostabilizers in these composites could deactivate the reactive products of PP degradation or consume the products of the initiation reaction, preventing their propagation [45].

### 3.2. Thermogravimetric Analysis (TGA)

Figure 3 shows the TGA curve and derivative thermogravimetry (DTG) curve of the systems before and after natural aging. Table 1 lists the decomposition temperatures (*T*_onset_, *T*_endset_, and *T*_dmax_) and the percentage of mass loss at 800 °C. The maximum degradation temperature was determined from the maximum peak temperature in the first derivative (TGA curve). After 120 days of weathering, all samples showed a reduction in all decomposition temperatures, suggesting that aging impacted the samples’ thermal properties.

The R-PP1x maintained higher thermal degradation temperatures, suggesting superior thermal stability even after weathering. Increasing the number of reprocessing cycles can introduce impurities, molecular degradation, and a reduction in the polymer’s thermal properties [46]. In contrast to the matrices, the composites exhibited reduced degradation temperatures. Polymers are susceptible to thermal damage, and constant exposure to high temperatures during each extrusion cycle can lead to thermal degradation. This degradation occurs when polymer chains break, resulting in a reduction in molecular weight. Previous studies have reported different behavior, where higher filler contents resulted in greater thermal stability [19,47].

The incorporation of CHF reduced the thermal stability of R-PP. In a polymer composite filled with particles, these particles are unlikely to be fully distributed and dispersed over the entire material area. Consequently, particle agglomeration was present. Therefore, these agglomerates can act as stress concentrators and weak points in the composite structure, and due to this, they can degrade more easily and quickly at high extrusion temperatures, which reduces thermal stability. Other factors can influence the result, such as interfacial compatibility between the formulation components, processing conditions, and thermal decomposition of the vegetable filler. However, this result may not negatively affect the mechanical properties of the composites, as the temperature range related to the greatest mass loss was similar for all samples because the matrix polymer remained thermally stable. A previous study showed similar results with different fibers (flax, jute, hemp, and sisal) [48,49,50,51].

Therefore, based on these results, it can be inferred that adding CHF up to a specific limit and additives can positively affect thermal stability before and after weathering, significantly influencing the amount of added filler. As in any preliminary scientific study, new replications and methodology optimization can better confirm the considerations raised here. Furthermore, new research must always be taken into account, including variations in the filler concentration, the search for methodologies to improve distribution and dispersion, possible surface modifications, and tests under other processing conditions, aiming to identify the filler’s more precise influence on the thermal stability of the PP-based composite.

Salazar-Cruz et al. [52] evaluated the thermal stability of PP composites filled with pistachio shells chemically treated with sodium hydroxide. The study confirmed that this chemical treatment effectively removed lignin and hemicellulose from the pistachio shell fibers, thereby increasing the temperature at which thermal decomposition begins. However, the remaining cellulose fractions accelerated the decomposition of the composite. Consequently, the authors suggested that the chemical treatment enhances the polymer-filler interaction. Therefore, the thermal stability of the analyzed composites with CHF fillers could be significantly improved using some chemical treatment to modify the interaction with PP. In other words, the chemical treatment of CHF can modify its surface characteristics, improving its compatibility with the PP polymer matrix and consequently obtaining more improved properties.

### 3.3. Mechanical Behavior

Table 2 lists the Young’s modulus, tensile strength, and elongation at break of the systems before and after natural aging, and their behavior is represented in Figure 4. When analyzing the behavior of the Young’s modulus, it was found that variations in composition and time of exposure to weathering significantly affected it (*p* < 0.05). In the absence of exposure to weathering, reprocessing significantly reduced Young’s modulus values by 14.4% (*p* < 0.05) between R-PP1x and R-PP3x. On the other hand, the presence of CHF generated a slight increase in Young’s modulus values, close to 10% (R-PP3x/5CHF) but not significant (*p* > 0.05). Finally, weathering reduced Young’s modulus values in all materials. However, each composition showed no significant difference in this property (*p* > 0.05). Only a maximum difference of 12% was observed for R-PP3x. Similar behavior was previously observed in PP/bamboo fiber composites during natural aging [19].

The tensile strength of the materials showed a significant difference similar to the Young’s modulus, that is, the composition and time of exposure to weathering significantly affected the results (*p* < 0.05), but their interaction (composition–time) did not. Before exposure to weathering, reprocessing did not significantly (*p* > 0.05) affect the tensile strength values, observing a slight reduction of less than 7%. CHF in the triple reprocessed matrix significantly increased the tensile strength values (*p* < 0.05), increasing 28.0% and 45.1% for R-PP3x/3CHF and R-PP3x/5CHF, respectively. Similar results were previously reported in polyester fiber/CHF composites [37].

After exposure, the tensile strength values were recorded as significantly different (*p* < 0.05), specifically between 0 and 120 days. Therefore, there was no significant difference (*p* > 0.05) between exposure times 0 and 30 days and 30 and 120 days. The variation in tensile strength values was between 4.2 MPa (R-PP1x) and 6.4 MPa (R-PP3x/5CHF). Other authors have also reported the loss of tensile mechanical properties in thermoplastic composites reinforced with natural fibers exposed to natural aging [53,54]. Widiastuti et al. [53] observed similar behavior when analyzing wood–plastic composites from recycled PP and industrial ironwood waste exposed to natural weathering. Differently, Alam et al. [17] reported that replacing a virgin PP matrix with a recycled matrix provides similar stability to photooxidative aging.

Variations in composition and time of exposure to weathering and their interaction (composition–time) significantly affected (*p* < 0.05) the elongation at break. Before exposure to weathering (zero days), triple reprocessing (R-PP3x) reduced the elongation at break values by 23.7% (R-PP1x). In comparison, the presence of CHF significantly reduced 80.1% and 82.7% for R-PP3x/3CHF and R-PP3x/5CHF (*p* < 0.05), respectively. Similar results were described in PP during multiple extrusions [54] and corn-starch-based hybrid composites reinforced with corn husks and palm fiber [55].

The exposure drastically affected the elongation at break values of the materials, reducing (*p* < 0.05) it by more than 88% in the compositions R-PP1x and R-PP3x from the initial 30 days. No significant differences (*p* > 0.05) were observed between the elongation at break values in these compositions after 30 days of exposure. The presence of CHF did not affect the elongation at break of the materials after exposure, so there was no significant difference (*p* > 0.05) between before and after exposure, confirming that the presence of CHF keeps elongation at break values significantly stable even under weathering conditions. Fajardo Cabrera de Lima et al. [19] reported that the elongation at break of PP and bamboo fiber composites was drastically reduced after six months of natural aging. Bais et al. [56] provided additional insights, emphasizing that heightened levels of ultraviolet radiation accelerate the degradation process, while climatic factors significantly influence the mechanical properties of natural composite materials.

Kuram [57] meticulously analyzed the effects of ultraviolet and thermal aging on PP composites reinforced with natural fibers (hazelnut shells, sunflower seed shells, and pumpkin seed shells). Their findings revealed that aging did not negatively impact the composite’s mechanical properties. Sunflower seed shell flour was the natural fiber that exhibited the best mechanical properties among those tested, further solidifying the findings and suggesting superior stress transfer capabilities.

### 3.4. Morphology Analysis

The morphology of exposed surfaces was evaluated using OM and SEM, as shown in Figure 4 and Figure 5. The degradation resulting from weathering primarily involves an oxidation process occurring on the polymer’s exposed surface, predominantly in non-crystalline regions. This process generates free radicals, leading to chain scissions [45,58].

The degradation mechanisms of PP composites with vegetable fillers involve photodegradation in PP and hydrolytic degradation of holocellulose, which are favored by environmental stress cracking [59] and climatic conditions [19,57]. The occurrence of chain scissions releases non-crystallized segments within the amorphous region, allowing them to reorganize into a crystalline phase. This results in contraction within the layers near the surface, compromising the mechanical properties and causing crack formation [58,60].

Analysis of the micrographs in the first 30 days of exposure to natural weathering revealed small lines on the surface of the R-PP1x system (Figure 4), with no changes in the other formulations. However, after 120 days, both reprocessed matrices showed cracks on their surfaces, indicating the occurrence of photodegradation (Figure 4 and Figure 5). In composite materials, discrete cracks were observed only in the composite with 3 wt% of CHF after 120 days but not in the composition with 5 wt% (Figure 4).

The composites exhibited diminished surface degradation during natural aging compared to polymer matrices. These measures shield the matrix from weather-induced effects and mitigate the impact of environmental conditions [45,60]. Notably, the phenomenon was less pronounced in R-PP3x/5CHF (Figure 5), which is characterized by higher fiber content and an increased presence of lignin, which contribute to the observed antioxidant effect [61,62,63,64,65].

Chemical treatments (such as alkaline treatment, silane, esterification, and isocyanate), physical treatments (such as plasma treatment), physicochemical treatments, and biological treatments can modify the surface of CHF. These modifications can significantly enhance the resulting composites’ performance [66,67].

## 4. Conclusions

Reprocessed PP composites were meticulously characterized, incorporating CHF, and subjected to natural environmental aging. Following a 120-day exposure period, the findings unveiled a pronounced degradation trend in the reprocessed matrices, manifesting in changes in morphological properties, chemical structure, and thermal characteristics. The presence of the selected vegetable filler helped preserve the PP, protecting it from photo-oxidative degradation due to the presence of lignin in the CHF. The mechanical properties exhibited a significant impact (*p* < 0.05), showcasing heightened brittleness and instances of catastrophic failures, both attributed to the presence and content of CHF. Contrastingly, composites featuring matrices reprocessed three times (R-PP3x/3CHF and R-PP3x/5CHF) demonstrated minimal property deterioration compared to their unexposed counterparts. This suggests that incorporating CHF yielded positive effects, enhancing stability against weathering. Consequently, these composites hold the promising potential to offer more cost-effective materials for the market and enhance their capacity to ensure a circular economy and manage agricultural waste. This promotes sustainability and contributes to the circular economy.

## Figures and Tables

**Table 1:** Thermal decomposition behavior of the systems (R-PP1x, R-PP3x, R-PP3x/3CHF, and R-PP3x/5CHF) before and after natural aging (0, 30, and 120 days).

Systems	Test Time (Days)	*T*_onset_–*T*_endset_ (°C)			*T*_dmax_ (°C)	ML * (%)
		1st Stage	2nd Stage	3rd Stage		
R-PP1x	0	390–468	-	-	446	100.0
	30	337–421	-	-	405	99.4
	120	327–436	-	-	418	99.4
R-PP3x	0	330–445	-	-	420	99.1
	30	330–424	-	-	403	100.0
	120	322–435	-	-	403	99.0
R-PP3x/3CHF	0	293–358	358–481	481–581	442	98.0
	30	304–376	376–451	451–479	404	99.9
	120	262–317	317–454	454–462	410	99.7
R-PP3x/5CHF	0	266–378	378–476	476–574	435	98.3
	30	283–345	345–442	442–449	390	99.6
	120	277–341	341–435	435–443	397	98.8

* Percentage of mass loss at 800 °C.

**Table 2:** Mechanical properties of the systems (R-PP1x, R-PP3x, R-PP3x/3CHF, and R-PP3x/5CHF) before and after natural aging (0, 30, and 120 days).

Systems	Test Time (Days)	Young’s Modulus (MPa)	Tensile Strength (MPa)	Elongation at Break (%)
R-PP1x	0	604.1 ± 9.0 ^a^	18.8 ± 1.6 ^b–d^	132.0 ± 25.0 ^a^
	30	561.8 ± 40.3 ^a,b^	16.5 ± 1.2 ^c–f^	12.0 ± 0.9 ^c^
	120	555.8 ± 20.5 ^a–c^	14.6 ± 1.6 ^e,f^	9.4 ± 1.1 ^c^
R-PP3x	0	516.9 ± 10.9 ^b–e^	17.5 ± 1.1 ^c–e^	100.7 ± 15.0 ^b^
	30	465.2 ± 21.4 ^e^	15.0 ± 0.8 ^d–f^	11.2 ± 1.0 ^c^
	120	454.9 ± 30.2 ^e^	12.9 ± 0.3 ^f^	8.1 ± 0.5 ^c^
R-PP3x/3CHF	0	537.1 ± 9.9 ^a–d^	22.4 ± 2.8 ^a,b^	20.0 ± 2.0 ^c^
	30	504.9 ± 20.1 ^b–e^	19.4 ± 1 ^b,c^	14.0 ± 0.8 ^c^
	120	488.8 ± 15.8 ^c–e^	17.2 ± 0.4 ^c–e^	10.3 ± 0.9 ^c^
R-PP3x/5CHF	0	541.1 ± 8.4 ^a–d^	25.4 ± 1.2 ^a^	17.4 ± 2.3 ^c^
	30	492.4 ± 30.9 ^b–e^	21.7 ± 1.5 ^a,b^	13.2 ± 0.4 ^c^
	120	481.6 ± 40.1 ^d,e^	18.9 ± 0.6 ^b,c^	9.9 ± 0.6 ^c^

Data are expressed as mean ± standard deviation (*n* = 7). The ANOVA indicated a significant difference between the means obtained. Different letters indicate a significant difference (*p* < 0.05) between the means using Tukey’s test with a 95% confidence level.

## Data Availability

Data are contained within the article.
